# Can CanMEDS competencies be developed in medical school anatomy laboratories? A literature review

**DOI:** 10.5116/ijme.5929.4381

**Published:** 2017-06-16

**Authors:** Joshua Hefler, Christopher J. Ramnanan

**Affiliations:** 1 Department of Innovation in Medical Education, Faculty of Medicine, University of Ottawa, Canada

**Keywords:** Medical education, anatomy, CanMEDS, competency-based, Canada

## Abstract

**Objectives:**

The purpose of this literature review was to identify potential ways in which undergraduate medical anatomy education may be relevant to the CanMEDS Roles, a competency-based framework used throughout Canadian medical training.

**Methods:**

A scoping review of medical education literature was conducted in March 2017 for English language publications that included key words related to anatomy education and to key competencies formally described for each of the Roles in the CanMEDS 2015 framework. Indicated benefits were then collated, characterized, and synthesized for each CanMEDS Role.

**Results:**

There were 71 studies identified describing original findings. Perceived benefits of anatomy education were most often identified for competencies related to the Medical Expert Role. Multiple studies also cited benefits related to the Scholar, Professional and Collaborator Roles. There was a lack of literature related to the Health Advocate, Communicator, and Leader Roles. The majority of benefits defined in the literature were limited to student perceptions rather than objectively measured outcomes.

**Conclusions:**

There is some evidence to suggest that anatomy education can facilitate the development of core competencies related to several CanMEDS Roles, outside of simply developing medical knowledge in the Medical Expert Role. Future studies need to develop methods to objectively assess outcomes related to these competencies.

## Introduction

CanMEDS is an educational framework created in the 1990s by the Royal College of Physicians and Surgeons of Canada to promote the development of essential skills amongst physicians in-training. Apart from a core Role as a Medical Expert, CanMEDS identified adjacent skills required by physicians, who aimed to provide the best possible patient care. The framework has been updated twice – in 2005 and just recently in 2015 – to reflect the changing realities of modern practice.^1^ Over the years, it has been adapted for use internationally, as well as at the undergraduate level throughout medical schools in Canada.^2^^,^^3^ Medical students are now expected to develop competencies related to the formal CanMEDs Roles of Medical Expert, Professional, Communicator, Collaborator, Leader, Health Advocate and Scholar.

Anatomy education has a longstanding history as a core basic science in the undergraduate medical curriculum. Over recent decades, however, there has been a substantial decline in the amount of time dedicated to anatomy teaching medical schools in several countries, including Canada.^4^^,^^5^ The length of medical school has remained the same (typically four years), but undergraduate medical education is now expected to develop medical student skills related to professionalism, ethics and the humanities, amongst other areas.^6^ Moreover, the trend for increased (and earlier) experiences in clinical contexts, including contact time with patients, has resulted in less time for basic sciences like anatomy. As curricular time is a finite, valuable resource in undergraduate medical education, the time- and cost-intensive (i.e. dissection-based laboratories) nature of anatomy education makes this particular basic science a frequent target for administrators looking for curricular time and space for new initiatives.^4^ A foundation of anatomical knowledge is required for the development of the Medical Expert Role in any physician.^4^ The depth required depends on one’s specialty, with surgery or radiology relying more on anatomical knowledge than other fields such as psychiatry or public health.^7^ Nonetheless, in addition to building clinically relevant knowledge of human structure, anatomy education in medical school may offer opportunities for the development of competencies related to a variety of CanMEDS Roles relevant across specialties. For example, the conventional approach to laboratory-based anatomy education typically involves students working in small groups around a cadaveric donor, which could facilitate the development of teamwork skills that are relevant to non-knowledge based competencies across several Roles.^5^

The purpose of this review article is to identify research linking anatomy education in medical school with the development of competencies related to the CanMEDS Roles. To our knowledge no such review of literature has been undertaken. The findings are intended to inform medical educators involved in anatomy curriculum reform and development, as well as identify gaps that may benefit from further research.

## Methods

This review of the literature used the established scoping review framework, as delineated by Arksey and O’Malley.^8^ We explored the literature addressing various elements of the CanMEDS Roles as they related to anatomy education for undergraduate medical students. Specifically, the literature was searched (as detailed below), studies were collated and their results were synthesized and interpreted on the basis of the authors’ expertise and in relation to our principle question of whether the undergraduate anatomy curriculum in medical schools does or can support the development of one or more of the CanMEDS Roles.

**Figure 1:**
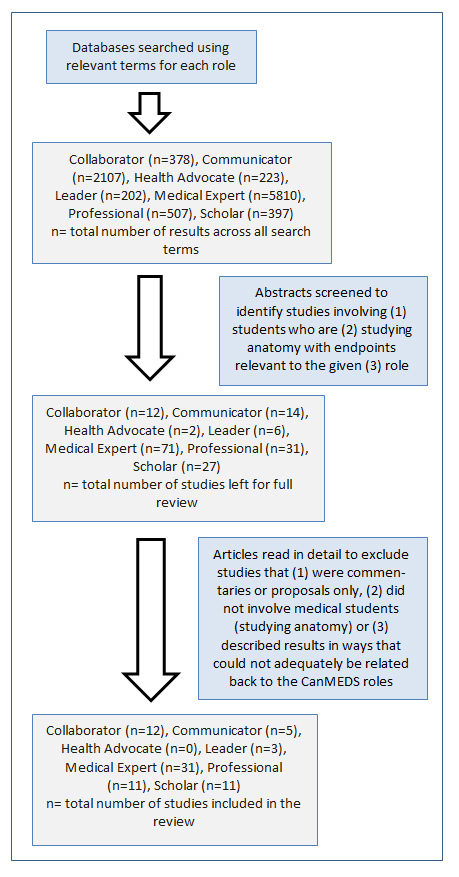
Schematic of the literature review process

Keywords were identified from the formal descriptions of the CanMEDS Roles from the Royal College of Physicians and Surgeons of Canada. Keywords were selected that were distinct to the particular Role.^1^ In addition to the names of Roles themselves, keywords included ‘clinical’, ‘decision’, ‘knowledge’, ‘reasoning’ for Medical Expert, ‘interprofessional’, ‘interdisciplinary’ for Collaborator, ‘empathy’ for Communicator, ‘community’ for Health Advocate, ‘involvement’, ‘responsibility’ for Leader, ‘ethics’, ‘accountability’, ‘behavior’ for Professional and ‘teach’, ‘research’ for Scholar. These keywords were utilized in combination with ‘anatomy education’ to search research publication databases, including PubMed, Google Scholar and Medline Plus (Figure 1). The search was completed on March 2017.

Abstracts were then identified (by primary author, J.H.) that linked anatomy education with a CanMEDS Role or a related, relevant defining term. In cases where search terms produce more than 10,000 results, the search was refined before abstracts were reviewed. At this stage, commentaries, proposals and studies not involving medical students were excluded. Studies that incorporated medical students in addition to other health science students (i.e. interprofessional education studies) were included. Both authors agreed upon the excluded articles and the reasons for excluding them.

Finally, the studies were read in detail to identify and summarize major conclusions. Both authors agreed on the inclusion of articles under specific CanMEDS Roles, based on their primary outcomes. All studies included original data, describing outcomes from an intervention involving medical students, within the context of anatomy education.

## Results

Overall, 71 different studies were identified, linking relevant features of CanMEDS Roles to anatomy education in medical school. None of the papers explicitly referenced CanMEDS, rather the outcomes studied were interpreted as being relevant to a particular Role based on the Royal College description of related competencies. The Medical Expert Role had the most papers identified, followed by the Collaborator, Scholar, Professional, Communicator, Leader, and Health Advocate Roles.

The majority of the 71 papers were purely descriptive in nature. The typical pattern consisted of the implementation of a particular approach, followed a description of the outcome. The most common method of evaluating outcomes was survey of student perceptions, which were used, to some extent, in 51 of the studies included. Several studies also combined this with peer assessment (2 studies) or objective measurements of skill or knowledge (14 studies). A minority of studies elaborated on this study design, either making comparisons between two cohorts or conducting their assessments pre- and post-intervention for comparison (4 and 8 studies respectively).

Six papers described studies comparing randomized groups of students. The largest contained 225 students, though it also included physiotherapy and physician assistant students in addition to medical students (81, 58 and 86, respectively).^9^ One randomized study containing only medical students had a total of 160 participants.^10^ On the opposite end, one study contained only 12 randomized medical student participants.^11^

### Medical Expert

The Medical Expert Role is the central CanMEDS Role that encompasses a physician’s scope of practice, incorporating their clinical knowledge and skills.^1^ Thirty-one studies were identified with this Role. Some addressed the Role of anatomy as part of students core knowledge, while others used the anatomy laboratory as a foundation for developing other clinical skills.

Three studies examined the structure of a school’s anatomy curriculum in relation to student performance on examinations and assessments. Generally, these studies determined that students performed similarly on assessments, regardless of modifications in the delivery of anatomy education. Bergman and colleagues looked at anatomy knowledge in medical students across 8 Dutch medical schools and found no correlation between the school’s approach to anatomy teaching and the student’s objective anatomical knowledge.^12^ Granger & Calleson and Marshak and colleagues tried to quantify the impact of cadaveric dissection on anatomy learning outcomes. Both describe the effects of a rotational dissection system, in which groups of students alternate between traditional cadaveric dissections and other methods of learning anatomy (e.g. video tutorials, anatomy texts).^11^^,^^13^ For both, there were limited, if any, differences in academic performance of those actively participated in dissection versus those that did not (i.e. learned that anatomy by alternative methods). These results are reasonably convincing, given the design of the studies, which compared cohorts and two randomized groups respectively. It must be noted that the latter study by Marshak and colleagues was limited by small medical student participant sizes (12 students in total).

The majority of studies relating to the Medical Expert Role (24 of 31) address the contribution of anatomy education to the development of related clinical knowledge or skills, including clinical reasoning and physical examination skills, surgical skills and ultrasound techniques (8, 7 and 6 studies respectively). One study specifically described the use of lightly embalmed cadavers to teach the Lachman test in conjunction with learning knee anatomy.^9^ The group randomly selected to learn on the cadavers showed both improved competence and confidence when performing the tests, though both groups had prior hands-on teaching. Three additional papers demonstrated that the integration of clinical skills, ranging from abdominal palpation to chest auscultation to musculoskeletal exams, into anatomy laboratories improved the learning of anatomical knowledge.^14^^-^^16^ Theses studies also make use of comparison groups, either cohorts or randomized, which adds to the strength of the evidence.

Seven studies described benefits related to the integration of surgical skills into the undergraduate medical anatomy laboratory. The surgical skills explored by the students varied from basic surgical skills, like knot tying, suturing, use of surgical instruments and cautery to working with laparoscopic instruments to even performing a full shoulder arthroplasty on a cadaver.^18^^-^^24^ Of these, all reported subjective benefits. Students’ perceived that the exercises were useful for their learning and development, and enjoyed the experience. Three studies looking at more objective benefits of surgically oriented skills had conflicting results.^19^^,^^23^^,^^24^ Nematollahi and colleagues showed an improvement in both clinical knowledge and technical skills after participation in a 2 hour, fresh cadaver-based emergency surgical skills laboratory.^19^ This was similarly demonstrated by Schoeb and colleagues over a semester-long course.^24^ In contrast, there were no differences found on assessments between a control group and a group that took a dissection course incorporating surgically oriented procedures on cadavers.^23^ Of note, only the latter study provided a comparison between students taking and not taking the course.^23^

Six studies described the introduction of ultrasound (US) into anatomy education, with varying effects on knowledge.^25^^-^^30^ Five applied US to in vivo models to complement anatomy teaching and introduce US to students.^25^^-^^27^^,^^29^^,^^30^ Of these, four brought US into the anatomy laboratory, in correlation with dissection topics, whereas the other held separate sessions outside of the lab that taught anatomy exclusively with US. The fourth study used US with cadavers to teach the anatomy and technique of several clinical procedures, including line insertion and pericardiocentesis.^27^ All of these studies described subjective benefits self-reported by students. Three studies also evaluated objective outcomes. In the study by Miller and colleagues, students’ knowledge improved throughout the course and the majority (83%) were able to obtain acceptable images.^30^ Dreher and colleagues similarly described increased student ability to identify key structures using US.^27^ Interestingly, Sweetman and colleagues reported a small but significant decrease in the performance of the US-instructed cohort on a relevant clinical assessment compared to the cohort that had not received US-guided teaching.^26^ Together, these studies show the potential for the successful implementation of US into the undergraduate anatomy curriculum in a variety of different ways, but consensus on their effectiveness is lacking.

### Collaborator

The essence of the Collaborator Role is the ability of physicians to work effectively with other healthcare professionals, with the unifying goal of providing the best possible care to patients.^1^ Twelve studies identified examined the effect of integrating medical students with students from other health professions around mutually relevant anatomy topics. The majority reported only survey results detailing students’ impression of the exercise. One study also included the observations of the session facilitators.^31^ Two other papers reported results across a semester long course and so was able to use grades as objective assessment of the impact of a multidisciplinary learning environment.^32^^,^^41^ There was uniform agreement on the benefits of this multidisciplinary approach across students from medicine, nursing, physiotherapy, radiography and biomedical science.

The results of the other studies generally support interdisciplinary activities as valuable experiences for medical students.^33^^-^^42^ Common themes that emerged include better understanding of the scope of other health care professions and enhanced mutual respect. Fernandes and colleagues even reported a positive benefit to the students’ professional identity, as individuals.^34^ Another group reported that having dental students involving in craniofacial anatomy actually improved the medical students’ motivation to learn and enhanced their perception of the importance of the subject matter in clinical practice.^37^ Only Krause and colleagues reported partially negative student perceptions of interprofessional anatomy education.^31^ They found the session involving 1st year medical and physiotherapy students were more collaborative, whereas the session involving 2nd year students took a more competitive tone. Furthermore, while both years valued and expressed appreciation for the session, the 2nd year medical students also voiced frustrations, feeling their knowledge of musculoskeletal anatomy and examination was inferior.

The majority (9 of 12) of interprofessional activities had students from different disciplines learning together. Two studies had the students from other health professions participating in the teaching, while McBride & Drake reported the results of an interprofessional activity that had medical students taking on the teaching Role with physician assistant students.^36^^-^^38^ Overall, these studies attest to the feasibility of integrating students of different healthcare professions in the anatomy laboratory, though further studies are warranted to assess their efficacy.

### Scholar

The Scholar Role emphasizes skills related to either research or teaching.^1^ The 11 studies identified here all relate to the development of teaching skills amongst medical students. In 10 of the 11 studies, this was achieved by giving students the opportunity to teach fellow medical students at (peer) or below (near-peer) their level. One study described medical students participating in continuing medical education for paramedics.^43^ All of the studies reported positive self-perceived benefits, with only one study reporting objective outcomes. Nnodim found that students participating in a peer-teaching program performed significantly better on their practical exam compared to a control group.^9^ A second study found similar academic benefits between groups of non-randomly selected students.^52^

Several studies focused on survey results from the students being taught.^44^^-^^46^ Duran and colleagues found no significant difference between student ratings of medical students and professors on dimensions of motivation, communication and performance.^44^ Similarly, Hall and colleagues found no significant difference in the quality of anatomy sessions taught by junior doctors compared to senior medical students, with students even reporting that they preferred the “delivery of the teaching” and experienced greater “enjoyment” in sessions given by medical students.^46^

Five studies reported the perspectives of the student-teachers.^47^^-^^51^ These papers presented a common theme of improved communication skills. Erie and colleagues showed further that students perceived development of competencies across several additional teaching domains, including course development and organization and student coaching and assessment.^50^ This study, conducted years after their participation in the student-teacher program, allowed these students to reflect on how it prepared them for the teaching demands of residency.^50^

Together, these studies demonstrate the feasibility of introducing medical students into the teaching role, with the anatomy lab as a backdrop. Though, this area of research would certainly benefit from studies to further elucidate the benefit of these exercises (i.e. whether students improve in their capacity as teachers).

### Professional

The physician’s Role as a Professional is to maintain certain standards of ethics and behaviour in providing care to patients, as well as being accountable for that care.1 Of the eleven studies identified, three describe the impact on students of interacting with family members of cadaver donors.^53^^-^^55^ The other studies looked at the ways in which the anatomy laboratory itself could contribute to the development of student professionalism.^56^^-^^63^

Talarico  described a program that promotes the view of the cadaver as the students’ “first patient”.^55^ They learn about the medical history of a body donor and their motivations for body donation, even receiving the cadaver from the funeral home and having to opportunity to meet with surviving family members afterwards. Student responses from the reflective component of the course report, among other things, that the increased interaction helped them better understand the emotions of the donor and their family, as well as develop a greater sense of compassion towards the cadaver. Kostas and colleagues and Crow and colleagues describe less intensive programs that similarly have students learning more about the cadaver they are dissecting through interactions with their families, also with positive results.^53^^,^^54^ Interestingly, Cahill & Ettarh found that students’ interest in donating their own bodies decreased from 31.5% to 19.6% after participating in a 9-week dissection course, with a similar decrease in having their family members donate from 31.7% to 14.7%.^57^

As to whether the gross anatomy laboratory itself contributes to the development of professional qualities, Pearson & Hoagland report an increase in altruism over a gross anatomy course.^60^ Other professional attitudes, including accountability, duty, excellence, integrity and respect, did not improve, but at least remained stable. Pawlina and colleagues found a positive correlation between personal qualities of integrity and responsibility and written and practical exam marks.^56^ Another study by Kuranakaran and colleagues served to highlight the role for anatomy educators in reinforcing professional behaviours and attitudes, including humanism, accountability and honesty.^63^ Two interesting studies propose interventions to develop students’ professionalism within the anatomy lab. One, by Shiozawa and colleagues, describe a professionalism seminar that ran concurrently with the anatomy course.^61^ A second, by Kissler and colleagues, introduced written reflections on professional themes as part of the anatomy course.^62^ Overall, these studies provide some evidence that the anatomy laboratory experience contributes to the development of professional attitudes.

### Communicator

Physicians as communicators are expected to develop the ability to share health information with patients.1 Of the five studies identified as relevant to developing Communicator competencies, only Evan directly links anatomy education with patient communication.^65^ This study had students design a leaflet on birth defects for patients, as part of a developmental anatomy course. Evaluations of the leaflets indicated a high degree of readability and accessibility to a wide patient audience. The others each employed techniques within the setting of the anatomy lab to utilize and/or develop student communication skills.^66^^-^^69^ Each describe a different method of engaging students, including oral examination, reflective essay writing, practicing patient handoffs and interacting on social media. They all reported positive student responses, with Hennessey and colleagues noting an effect on exam marks as well.^69^ While these studies do not provide strong evidence for any particular technique, they do demonstrate the feasibility of these initiatives and offer hypotheses for further research.

### Leader

The Royal College identifies Leaders as those who can “contribute to a vision of a high quality health care system” by engaging others and taking responsibility for delivering quality patient care.^1^ Few studies have identified leadership competencies being enhanced in anatomy education. Of the three studies identified, two were also identified being relevant to competencies in other Roles. Krych and colleagues mentioned the development of leadership skills in their description of a reciprocal peer teaching exercise (described under the Scholar Role).^48^ Pawlina and colleagues did look at different leadership styles amongst medical students in the context of small groups within a gross anatomy (in addition to characteristics related to professionalism).^56^ They did not look at a relationship between anatomy and leadership, but were able to identify styles of leadership best suited to small group anatomy learning. One report described a project where medical students in a gross anatomy course construct a case from a given diagnosis for written and oral presentation.^64^ The authors claimed that this approach developed teamwork and leadership skills, but these skills were not formally assessed. There is certainly room for further exploration in this area, but it is perhaps best done in conjunction with other Roles.

### Health Advocate

Physicians as health advocates are expected to use their expertise and influence to address the needs of a particular group.^1^ In our search, no studies were found that connected anatomy education with competencies of this Role.

## Discussion

While no study directly mentioned CanMEDS, many diverse studies were identified that link anatomy education with a key competency of a CanMEDS Role. These studies provide examples of ways in which anatomy education can support the development of the modern physician, regardless of specialty, outside of providing clinical anatomical knowledge. Overall, many of the studies reviewed described innovative educational approaches that were well-received by students.

Studies that were identified as relevant to the Medical Expert Role largely supported the development of clinical skills, in conjunction with anatomy education. These varied from physical exam skills to surgical skills to acumen with ultrasound technology.^15^^,^^16^^,^^18^^-^^30^ Taken together, this studies provide extensive support for the integration of clinical skills in medical anatomy education, both in terms of student learning and interest. The number of studies found under this role is unsurprising, given the close relationship between these skills (i.e. relating internal organs to surface anatomy) to basic anatomical knowledge. The use of ultrasound is particularly noteworthy, with its rising popularity amongst clinicians.^70^^,^^71^ As such, future integration of ultrasound into medical anatomy education may reinforce the importance of anatomy in clinical settings.

Multiple studies were also identified as being relevant to the Collaborator, Scholar and Professional Roles. In the case of the Collaborator and Scholar Roles, the studies described variations on a common theme. With the former, studies showed how anatomy sessions can facilitate interaction of medical students with diverse allied healthcare students.^31^^-^^42^ With the latter, it was medical students as teachers – of each other, of junior medical students and of other health professionals – centred around anatomy education.^44^^-^^52^

Few studies were identified as being relevant to the Communicator and Leader Roles. Furthermore, most of these studies related to a different component of each role. The results of these studies do not provide much practical guidance, but they do suggest topics for further exploration. For example, looking at the ways interacting with donors’ families can develop compassion or empathy, and maintaining these traits in current medical trainees is widely considered to be a concern.^50^^,^^58^  Moreover, no studies were identified that related anatomy education to competencies related to the Health Advocate Role. This is unsurprising, as the Health Advocate Role is entirely patient-centred and the only patients students have a chance to interact with are those who have donated their bodies after death. There may be potential to develop this role in the context of anatomy education by involving students in promoting and educating the public on body donation, but the relevance of anatomy education to Health Advocate-related competencies appear to be limited.

One major limitation of the studies identified is that the majority (nearly 90%) of were purely descriptive. Studies comparing randomized samples (that provide a stronger degree of evidence for a particular intervention) were relatively infrequent. As well, the majority of studies reported outcomes that were quite subjective, including many (~70%) only reporting perceived benefits and improvements by students, as objective outcomes were limited across the papers reviewed. A second important limitation is that none of the studies directly mention the CanMEDS roles. While the competencies described by the CanMEDS framework are almost universally desired in modern physicians, the formal CanMEDs descriptions are not, themselves, formally used in accreditation or medical program design in the majority of institutions worldwide.

## Conclusions

While this literature review yielded many studies  suggesting that anatomy education can facilitate the development of competencies relevant to the formal CanMEDS Roles, the majority of studies to date tend to be descriptive in nature, feature relatively weak evidence (i.e. few randomized control designs or objectively measured outcomes), and are typically reliant on subjective surveys of student perceptions. While evidence for the benefits of anatomy education to developing Professional, Communicator, Leader, and Health Advocate competencies appear particularly limited, there does to be evidence to suggest that medical anatomy education can help develop competencies related to the Medical Expert (clinical skills), Scholar (teaching skills), and Collaborator (interprofessional skills) Roles. While future studies should be designed to provide stronger evidence for these benefits, it does appear that medical anatomy education can provide a context for the development of valuable competencies in medical students outside of simply improving anatomical knowledge. These benefits should be kept in mind by medical students, medical educators, and program administrators when contemplating whether anatomy education should be maintained or reduced in modern medical curricula.

### Conflict of Interest

The authors declare that they have no conflict of interest.
